# Promiscuity of the *Euonymus* Carbohydrate-Binding Domain

**DOI:** 10.3390/biom2040415

**Published:** 2012-10-06

**Authors:** Elke Fouquaert, Els J.M. Van Damme

**Affiliations:** Laboratory of Biochemistry and Glycobiology, Department of Molecular Biotechnology, Ghent University, Coupure links 653, 9000 Ghent, Belgium; Email: elke.fouquaert@ugent.be (E.F.)

**Keywords:** binding site, carbohydrate, cytoplasm, lectin, nucleus, specificity

## Abstract

Plants synthesize small amounts of carbohydrate-binding proteins on exposure to stress. For example, on exposure to drought, high salt, wounding and by treatment with some plant hormones or by pathogen attack. In contrast to the ‘classical’ plant lectins that are mostly located in the vacuolar compartment, this new class of inducible lectins is present in the cytoplasm and in the nucleus. Taking into account that any physiological role of plant lectins most likely relies on their specific carbohydrate-binding activity and specificity, the discovery of these stress-related lectins provides strong evidence for the importance of protein-carbohydrate-interactions in plant cells. Hitherto, six families of such nucleocytoplasmic lectins have been identified in plants. This review will focus on the nucleocytoplasmic lectins with one or more *Euonymus* lectin (EUL) domain(s). The carbohydrate-binding specificity of EUL proteins from a monocot, a dicot and a lower plant has been compared. Furthermore, modeling of the different EUL domains revealed a similar ß-trefoil fold consisting of three bundles of ß-sheet organized around a pseudo three-fold symmetry axis. Despite the sequence similarity and the conserved amino acids in the binding site, glycan array analyses showed that the EUL domain has a promiscuous carbohydrate-binding site capable of accommodating high mannose *N*-glycans, blood group B related structures and galactosylated epitopes.

## 1. Introduction

Lectins are carbohydrate-binding proteins that recognize and bind well-defined simple sugars or more complex carbohydrates in a reversible way. These carbohydrate-binding domains are widespread but have been studied most intensively within the plant kingdom. Plant lectins are a very diverse group of proteins with obvious differences in their biochemical/physicochemical properties, molecular structure, carbohydrate-binding specificity and biological activities [1]. Although plant lectins have been known since the 19^th^ century, many questions regarding their physiological importance remain unanswered. 

In the early days of lectinology, research focused on those plant lectins expressed constitutively in high concentrations of seeds and in vegetative storage tissues. Most of these lectins are synthesized on the endoplasmic reticulum and finally end up in the vacuolar compartment of the cell. Biochemical and molecular studies of numerous lectins demonstrated that only a limited number of carbohydrate-binding motifs are present in plants. Furthermore some glycans are recognized by structurally unrelated plant lectins [2]. Since many of the abundant classical plant lectins bind to complex animal *N*- and *O*-glycans the idea was gradually developed that most of these lectins represent a special class of aspecific defense proteins that help the plant to cope with attacks from phytophagous invertebrates and/or herbivorous animals. This concept is further supported by the high expression levels of most lectins (generally 0.1–10% of the total protein), their accumulation in a developmentally regulated manner and the toxicity of multiple lectins for insects and fungi [3]. This rigidly preprogrammed expression implies that these lectins are not part of a response of the plant to a specific environmental stimulus. To explain the fairly widespread occurrence of these abundant lectins in seeds as well in different storage organs it is suggested that they combine a defense-related role with a function as a storage protein. Thus plants accumulate large quantities of lectins normally acting as storage proteins that, whenever appropriate, can also be recruited for defense purposes [2,4]. 

During the past 10 years, evidence has accumulated that some plant species synthesize well-defined carbohydrate-binding proteins upon exposure to stress situations such as drought, high salt, wounding, by treatment with some plant hormones or pathogen attack [5,6,7]. These lectins are present in low but physiologically relevant concentrations and are exclusively expressed in the cytoplasm and/or nucleus of the plant cell, and therefore are called nucleocytoplasmic lectins [8]. Based on these observations the concept was developed that lectin-mediated protein-carbohydrate interactions in the cytoplasm and the nucleus play an important role in the stress physiology of the plant cell [9,10,11]. 

Hitherto, six families of nucleocytoplasmic lectins have been identified in plants [8]. The identification of these nucleocytoplasmic lectins puts the physiological role of plant lectins in a new perspective and indicated that at least some plant lectins interact, like many animal lectins, with endogenous glycan receptors. Although there is good evidence for the carbohydrate-binding properties of at least some of the inducible lectins, there are at present few indications for the possible receptors for these lectins inside the plant cell. For example, the jasmonate-inducible tobacco leaf lectin (referred to as Nictaba) locates to the cytoplasm and the nucleus of tobacco parenchyma cells, and can interact in situ with *N*-glycosylated nuclear proteins [12]. Recently it was also shown that this lectin can interact with *O*-GlcNAc modified histone proteins inside the nucleus. Since this lectin-histone interaction was shown to be carbohydrate dependent, it was suggested that Nictaba fulfills a signaling role in response to stress by interacting with *O*-GlcNAcylated proteins in the plant cell nucleus [13].

This review will focus on the family of nucleocytoplasmic lectins grouping all proteins that show homology to the *Euonymus* lectin (EUL). An overview of the occurrence, carbohydrate-binding properties and three-dimensional conformation will be presented and discussed in view of the putative physiological role of these so-called EUL-related lectins in the plant.

## 2. Lectins with an EUL Domain

In 2008 a new family of nucleocytoplasmic lectins comprising all proteins that contain at least one *Euonymus* lectin (EUL) domain was identified. This EUL domain was shown to represent a conserved structural unit of a novel family of putative carbohydrate-binding proteins [14]. Although it was known for a long time that the arillus tissue of spindle tree (*Euonymus europaeus*) contains very high concentrations of the so-called *Euonymus europaeus* agglutinin (EEA) [15,16,17], this lectin could not be classified into any of the known lectin families due to lack of sequence information. 

### 2.1. Molecular Cloning of EEA

Molecular cloning and sequencing of the lectin cDNA demonstrated that the EEA subunits contain 152 amino acid residues, encoding a polypeptide of approximately 17 kDa. Two subunits form the 37 kDa homodimeric non-glycosylated lectin. Since no putative signal peptide could be identified in the deduced sequence of the lectin cDNA it was hypothesized that EEA is synthesized on free ribosomes [14]. Confocal microscopy of tobacco cells, expressing GFP-fusion constructs with EEA, confirmed the localization of the protein in the cytoplasm and the nucleus of the cells [18]. 

Sequence comparisons indicated that the EEA sequence did not show sequence similarity with any other lectin. Therefore it was not possible to classify EEA into one of the known lectin families. However, the EEA sequence shares a high sequence similarity (62%) with a domain that was identified in some abscisic acid and salt-stress responsive rice proteins which presumably plays a role in the adaptation of the roots to a hyperosmotic environment, referred to as OSR40 proteins [19]. These rice proteins are annotated in the database as “Ricin-B related lectin domain containing proteins” based on the presence in their sequence of two QXW repeats, which are considered typical motifs of the ricin-B domain. However, taken into account the low sequence identity/similarity between the amino acid sequences of the OSR40 proteins and the ricin sequence it is inappropriate to classify these proteins in the ricin-B family [14]. Therefore EEA and the OSR40 proteins are now classified in a new family of so-called proteins with EUL domain(s).

### 2.2. Occurrence of Plant Proteins Containing an EUL Domain

Screening of the publicly accessible databases revealed that proteins with an EUL domain are ubiquitous within the Embryophyta, but are not present in other eukaryotes or in prokaryotes [5]. At present EUL sequences have been found in monocots such as maize, rice and *Sorghum*, in dicots such as *Arabidopsis*, tomato and poplar, but also in lower plants such as the mosses *Physcomitrella*, *Selaginella* and *Marchantia*. The widespread distribution of the EUL domain strikingly contrasts the more limited or even narrow distribution of most other lectin domains found in plants [8]. 

Nevertheless there is some heterogeneity within the EUL family. Some EUL proteins are hololectins, which are exclusively composed of carbohydrate-binding domains (such as the *Euonymus* lectin). However, the majority of the EUL sequences encode chimerolectins, since they consist of one or more carbohydrate-binding EUL domain(s) arrayed in tandem to a non-related *N*-terminal domain that varies in length from 10 to 200 amino acids. Examples of chimerolectins can be found in rice, *Arabidopsis* and *Physcomitrella*. Both the *N*-terminal domain and the linker sequences between EUL domains are highly variable, whereas the sequence of the EUL domain itself is fairly well conserved. 

**Figure 1:**
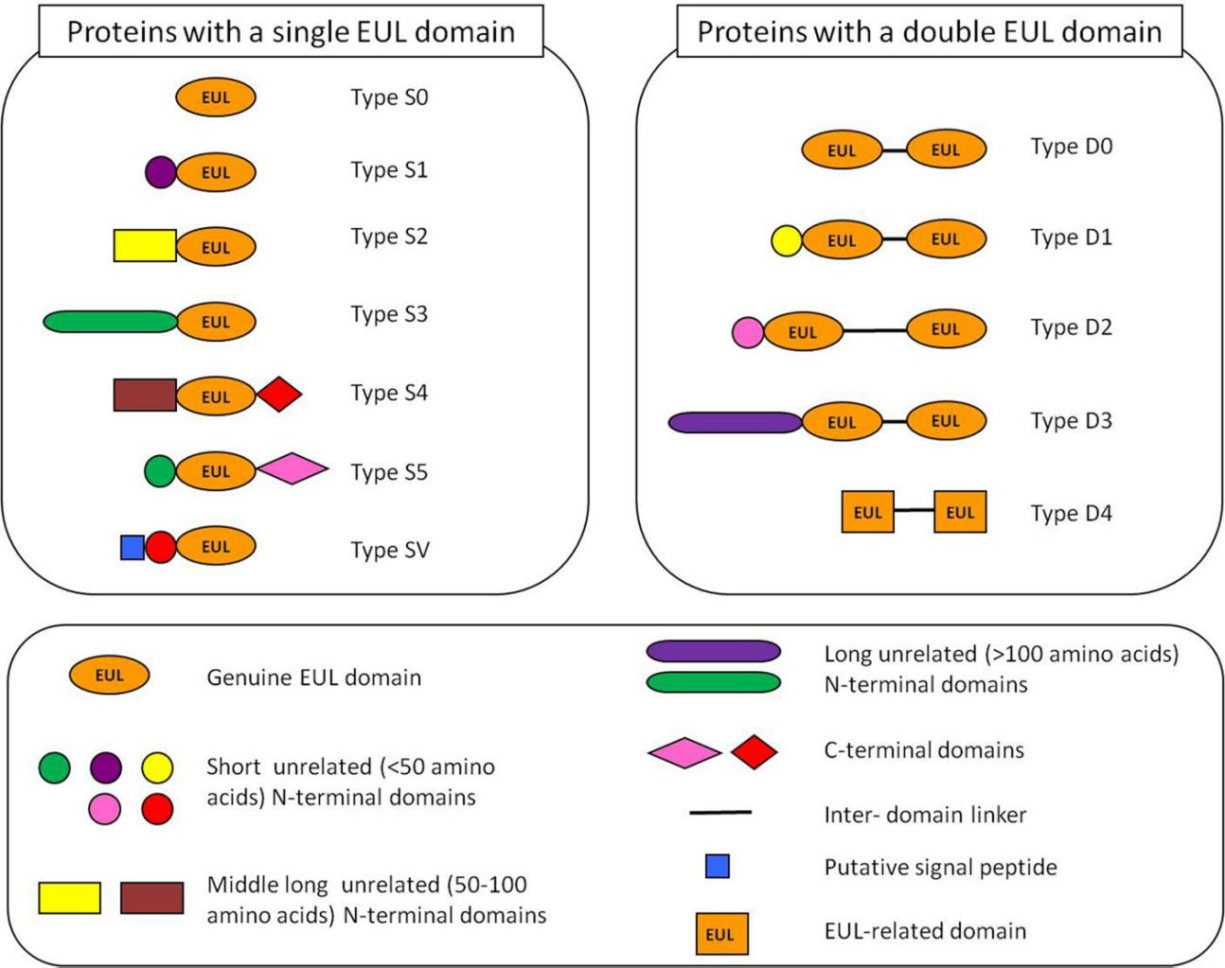
Schematic representation of the 12 types of *Euonymus* lectin (EUL) proteins found in Embryophyta. The S0, S2 and S3 types were analyzed in more detail in our study.

Based on the overall domain architecture of all EUL sequences known to date a classification system for this lectin family was proposed [5]. EULs can be classified in 12 types (Figure 1). Basically, the EUL family can be subdivided into single and two-domain proteins, containing one or two EUL domains, respectively. The proteins that consist exclusively of EUL domains (like EEA) are classified as the type S0 EUL proteins. However, in most cases an unrelated *N*-terminal domain with variable sequence precedes the EUL domain. Depending on the length of this *N*-terminal domain three different types of EUL proteins can be distinguished, being a short (<50 amino acids, type S1), medium long (50–100 amino acids, type S2) or long (>100 amino acids, type S3) *N*-terminal domain (Figure 1). In addition to the *N*-terminal domain, an additional domain with variable length can also be present at the *C*-terminus of single-domain EULs (type S4 and S5, Figure 1). The two-domain EUL protein sequences are classified into 4 types based on the length of the *N*-terminal domain preceding the EUL domains and the linker sequence between the two EUL sequences (type D0, D1, D2 and D3) (Figure 1). 

A detailed analysis of the EUL sequences in dicot plants revealed that most dicot species such as *Arabidopsis* only have one or two genes encoding a single domain EUL sequence with a long unrelated *N*-terminal domain (type S3). In contrast, monocot and lower plant species have a whole set of EUL sequences encoding single as well as two-domain EUL proteins. For instance, in rice four different genes encoding EUL proteins are present. Moreover, lower plants also have different types of EUL proteins that cannot be found in higher plants, such as chimerolectins that contain next to the EUL domain a *C*-terminal domain [5]. In some lower plants such as in *Selaginella moellendorfii* EUL proteins were found that consist of two in tandem arrayed EUL-related domains separated by a short linker (Figure 1, type D4). However, the EUL-related domains of these proteins share only a low sequence similarity with the genuine EUL domains. The S3 type of EUL sequences was shown to be present in most if not all *Viridiplantae*, in dicots, monocots and lower plants, suggesting an important role for this universal type of EUL proteins. Moreover, these S3 type EUL proteins are encoded by genes with a strictly conserved intron/exon structure [5].

### 2.3. Expression of Plant Proteins Containing an EUL Domain

Analysis of the EUL sequences revealed that most EULs are synthesized without a signal peptide, and therefore will encode cytoplasmic proteins. However, in some monocots and lower plants single domain EUL proteins with a putative signal peptide have been identified suggesting that some vacuolar EULs could exist (Figure 1, type SV). It should be mentioned, however, that until now we did not succeed in amplifying any of these EUL sequences with a signal peptide. Confocal microscopy of transformed BY-2 or *Arabidopsis* cells expressing EGFP fusion constructs demonstrated that the S3 type EUL protein from *Arabidopsis* and rice are located in the cytoplasmic and nuclear compartment of the cell, as shown for EEA [18,19].

As already mentioned above, the rice EUL proteins, previously referred to as OSR40 proteins, are induced by different stresses. However, it remains to be shown if all proteins containing one or more EUL domains are also involved in the adaptive response to a hyper-osmotic environment, as suggested for some EUL proteins from rice [20]. The upregulation of the rice EUL transcripts after salt-stress was also observed during investigations with microarrays including 1728 cDNAs from libraries of salt-stressed roots. Transcripts identical to the OrysaEULD1B protein showed a peak expression in the salt-tolerant rice variety Pokkali and the salinity-sensitive rice variety IR29 after 3h of salt-stress. An upregulation of OrysaEULD1A was only observed after 24h of salt stress [21]. Next to roots and shoots, the EUL proteins were also identified in panicles. Proteomics of young rice panicles revealed that the protein OrysaEULS3 was up-regulated 4-fold in response to salt stress [22]. In 2009, proteomics approaches revealed that the putative r40c1, which corresponds to the S3 type EUL protein from *Oryza sativa,* was dramatically dephosphorylated during drought stress [23]. More evidence for the fact that EUL proteins are associated with stress adaptation was given by the identification of an EUL homolog in maize (*Zea mays*) [24]. Increased protein levels of this maize EUL homolog were found in the leaves when maize plants were subjected to a water deficit [25]. Two isoforms of the EUL family were also identified in banana (*Musa* spp.) [26]. In shoot meristem cultures of banana, EUL expression was slightly upregulated after high sucrose stress. In the dehydration tolerant variety EUL was a very abundant protein under high concentrations of sucrose, while the expression level of EUL in the dehydration-susceptible variety is 6.4-fold lower [27]. It was put forward that EUL proteins might contribute towards dehydration tolerance [27]. Furthermore Moons *et al*. [20] suggested that some rice EUL proteins play an important role in the response of plant tissues to salt and osmotic stress. An *in silico* expression analysis for the EUL from *Arabidopsis* demonstrated that this lectin gene is upregulated by salt-stress and osmotic stress and upon treatment with abscisic acid [5]. 

Judging from these data, it can be concluded that EEA is the prototype of a novel family of stress-inducible cytoplasmic/nuclear proteins, all containing at least one so-called EUL domain [14]. The apparent omnipresence of the EUL domain is indicative for a universal role of this lectin domain in plants. Taking into consideration that EUL proteins are located in the cytoplasmic/nuclear compartment, and, in addition, are involved in responses to stress, the identification of this new family of lectins led to the hypothesis that lectin-mediated protein-glycoconjugate interactions are essential for some important cellular processes in Embryophyta [9,10,11]. It is hypothesized that EUL proteins can help the plant to cope with a lot of environmental stresses and improve the stress tolerance in plants. 

### 2.4. Analysis of EUL Proteins from Different Origins

Although all EUL domains show highly conserved amino acid sequences this does not necessarily imply that all proteins have carbohydrate-binding activity and will recognize similar glycan structures. Therefore three single-domain EUL sequences from different origin have been selected and investigated for their carbohydrate-binding properties and the conformation of the binding site.

In this review a comparative analysis will be made of the sequence, carbohydrate-binding properties and three-dimensional conformation of EEA and the EUL domains in the S3 type EUL proteins from the dicot *Arabidopsis thaliana* (further referred to as ArathEULS3, At2g39050) and the lower plant *Physcomitrella patens* (PhypaEULS3, JGI: scaffold_74 (415096:416617), and the S2 type EUL protein from the monocot *Oryza sativa* (Os07g0684000, OrysaEULS2).

**Figure 2:**
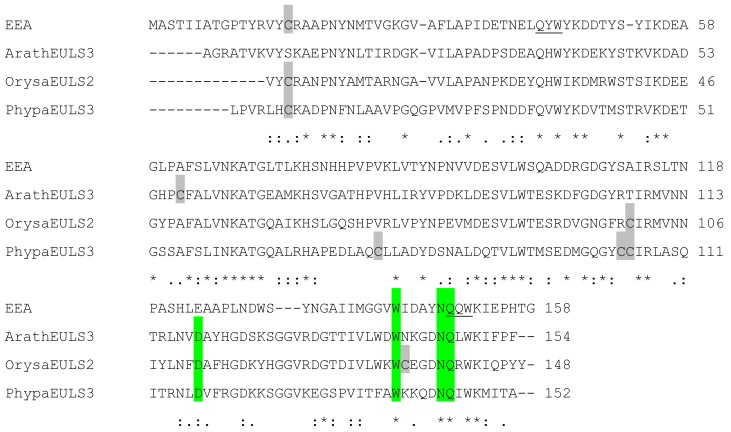
Sequence alignment of the amino acid sequences encoding the EUL domains from *Euonymus europaeus* (EEA), *Arabidopsis thaliana* (ArathEULS3), *Oryza sativa* (OrysaEULS2) and *Physcomitrella patens* (PhypaEULS3). Identical residues are indicated by asterisks and similar residues by dashes or colons. The amino acids that form the putative carbohydrate-binding site are shaded green. The cysteine residues are indicated in grey. The QXW motifs are underlined in the EEA sequence.

Analysis of the EUL sequences ArathEULS3, PhypaEULS3 and OrysaEULS2 revealed that the EUL domains from these different plant species showed 25% sequence identity and 57% sequence similarity to the EUL domain of EEA (Figure 2). The highest percentage of identity was observed for sequences from ArathEULS3 and OrysaEULS2 (63% identity, 86% similarity), whereas EEA and PhypaEULS3 showed less homology (33% identity, 71% similarity). Although the sequences of the EUL domains are conserved, little sequence homology is observed among the *N*-terminal domains of the S3 proteins from *Arabidopsis* (163 AA) and *Physcomitrella* (124 AA), and the S2 type sequence from rice (56 AA). Moreover, these unrelated *N*-terminal domains share no significant similarity with any other known domain. Some of these *N*-terminal domains are rich in histidine residues such as the *N*-terminal domain of ArathEULS3 and PhypaEULS3, but this is not the case for OrysaEULS2. 

A comparative analysis of the carbohydrate-binding properties of the EUL domains requires the purified proteins. Unfortunately, their low expression levels hamper the identification and characterization of most of the EUL proteins. In contrast to EEA, which is expressed at relatively high levels in the arilli of the spindle tree seeds (500 µg/g of dry arillus material), the EUL proteins from *Arabidopsis*, rice and *Physcomitrella* are very low abundance proteins. Therefore the EUL domains of ArathEULS3, OrysaEULS2 and PhypaEULS3 were recombinantly expressed in the heterologous expression system *Pichia pastoris* and the recombinant proteins purified [18,28].

## 3. Carbohydrate-Binding Properties of the EUL Domain

The carbohydrate specificity of EEA and of the EUL domains of the S3 type EUL protein of the dicot *Arabidopsis thaliana* (ArathEULS3), the S2 type of the monocot *Oryza sativa* (OrysaEULS2) and the S3 type of the lower plant *Physcomitrella patens* (PhypaEULS3) was analyzed first by agglutination assays and by inhibition of cellular agglutination using a panel of monosaccharides and glycoproteins. Subsequently, more detailed analyses of the carbohydrate-binding properties were performed using the glycan array technology developed by the Consortium for Functional Glycomics, which enables high throughput screening of large collections of carbohydrates and more complex glycans with only a small amount of a purified lectin [29,30].

### 3.1. Agglutination and Inhibition Assays

The agglutination properties of the different EUL proteins were determined using agglutination assays with rabbit erythrocytes. EEA readily agglutinates the red blood cells, the minimal concentration of EEA required for agglutination being 1.7 µg/mL [31]. Recombinant OrysaEULS2 and PhypaEULS3 were also able to agglutinate the red blood cells only after 30 min, but semi-quantitative agglutination assays indicated that these recombinant proteins OrysaEULS2 and PhypaEULS3 required considerably higher concentrations of the protein, the minimal concentration for agglutination being 54 µg/mL and 92 µg/mL, respectively. In contrast the recombinant ArathEULS3 showed very low if any agglutination activity. The agglutination results suggest that –like EEA- the other EUL lectins under study are multimeric proteins, which is in agreement with the preliminary results of a gel filtration analysis.

Inhibition assays with a series of simple sugars indicated that EEA purified from the arilli was best inhibited by the monosaccharide mannose and the disaccharide lactose. Of all glycoproteins tested thyroglobulin was the most potent inhibitor (EC50 value 250 µg/mL) for EEA, scoring better than asialofetuin and asialomucin (Table 1). These data are in line with the results of inhibition assays performed with human type O erythrocytes by Petryniak *et al*. [16] who showed that very high concentrations of lactose (11 mg/mL) can inhibit the agglutination of EEA. Further studies with milk and blood group oligosaccharides revealed that the lectin most specifically reacts with blood group B substances and to a lesser extent with blood group H substances [16]. 

The agglutination of rabbit erythrocytes by the recombinant OrysaEULS2 protein from rice was best inhibited by mannose, methyl α- mannopyranoside and lactose as well as the glycoproteins thyroglobulin, ovomucoid and asialomucin [28]. In contrast mannose and methyl α-mannopyranoside did not inhibit the agglutination of rabbit erythrocytes by the recombinant PhypaEULS3 from the lower plant *Physcomitrella*. Galactose, lactose and the glycoproteins thyroglobulin, asialomucin and mucin were potent inhibitors of the agglutination caused by PhypaEULS3 (Table 1). 

**Table 1:** Carbohydrate-binding specificity of EEA, the EUL domain of OrysaEULS2 and the EUL domain of PhypaEULS3 determined by agglutination inhibition assays with differentsugars or glycoproteins. - : no inhibition of agglutination; + weak inhibition of agglutination; ++ strong inhibition of agglutination; +++ very strong inhibition of agglutination.

	EEA [15,16,31]	EUL domain of OrysaEULS2 [28]	EUL domain of PhypaEULS3
**Sugar**			
Mannose	+++	+++	-
Methyl α-mannopyranoside	Not tested	++	-
Galactose	-	-	++
*N*-acetylglucosamine	-	-	-
Arabinose	-	Not tested	Not tested
Glucose	-	-	Not tested
Fucose	-	Not tested	Not tested
Glucosamine	-	Not tested	Not tested
Lactose	++	++	++
**Glycoprotein**			
Thyroglobulin	+++	+++	+
Ovomucoid	+	++	-
Asialomucin	+	+++	++
Mucin	-	-	++
Fetuin	+	-	-

### 3.2. Glycan Array Analysis

**Figure 3:**
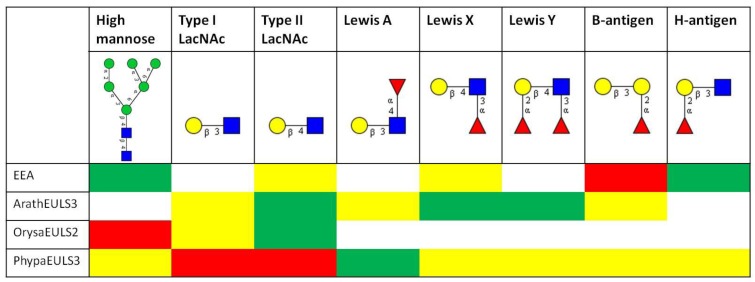
Comparative analysis of the glycan array binding of EEA and the recombinant EUL domains of the S3 type EUL protein from *Arabidopsis* (ArathEULS3), the S2 type EUL protein from *Oryza sativa* (OrysaEULS2) and the S3 type protein from *Physcomitrella patens* (PhypaEULS3). For each lectin the top 30 glycans with highest reactivity on the glycan array were analyzed for the presence of the following carbohydrate motifs: high mannose *N*-glycans, type 1 and type 2 LacNAc motifs, Lewis A, Lewis X, Lewis Y, B-antigen and H-antigen structures. Based on the frequency of these structures in the top 30 glycans the percentage of relative fluorescence units (%RFU) for each glycan was calculated and different colors have been assigned: red = structure is frequently present and glycans with this structure have a high %RFU and strongly bind with the lectin; green = structure is present but glycans with this structure have a lower %RFU; yellow = glycans with these structures are not frequently present in the top 30 glycan structures and weakly bind to the lectin; white = glycans with these structures are not recognized by the lectin. The different glycan structures are represented using the glycan symbol nomenclature used by the Consortium for Functional Glycomics [67].

To get a better insight into the sugar-binding specificity of the EUL domain and to get more quantitative data, the proteins were also analyzed on the glycan array, and a comparative analysis of the data obtained for EEA, the recombinant EUL domain from ArathEULS3, OrysaEULS2 and PhypaEULS3 was made. For each lectin the top 30 glycan structures with highest reactivity on the glycan array were analyzed for the presence of different carbohydrate motifs (Figure 3). 

Detailed analyses of the interaction of the fluorescently labeled EEA with the array revealed a high affinity of EEA especially for glycans containing the B-antigen structure, but also other blood group B-related structures such as glycans containing the H-antigen structure are recognized by EEA. These data are in agreement with earlier results that proved high affinity of EEA for Galα1-3Gal and Galα1-3Galß1-4GlcNAc carbohydrate epitopes, and binding to the blood group B (Galα1-3[Fucα1-2]Gal-) and O epitopes (Fucα1-2Gal-) as well as some types of glycosphingolipids [17,32,33]. Our reinvestigation of the carbohydrate-binding specificity of EEA using a glycan array screening confirmed the previously reported high reactivity of EEA towards blood group B oligosaccharides but in addition also revealed binding of EEA to high mannose *N*-glycans (Figure 3). It was shown that the binding of EEA towards *N*-linked glycans requires the core pentasaccharide [Manα1,3(Manα1,6)Manß1,4GlcNAcß1,4GlcNAc]. Binding assays carried out at different lectin concentrations allowed the conclusion that EEA has a much higher affinity for blood group B oligosaccharides than for high mannose *N*-glycans [14]. In an attempt to determine whether EEA possibly possesses two independent binding sites which would allow interaction of EEA with structurally unrelated carbohydrate structures the glycan array screening experiment was also performed in the presence of inhibitory oligosaccharides for high mannose *N*-glycans and for the blood group B substances. However, no final conclusion could be drawn with respect to the possible occurrence of two distinct carbohydrate-binding sites in EEA [14].

The glycan array data for the recombinant ArathEULS3 from *Arabidopsis* revealed that this EUL lectin interacts preferably with complex *N*-glycans, since especially binding to galactosylated structures was observed. ArathEULS3 recognizes glycans containing one or more Lewis X (Galß1-4(Fucα1-3)GlcNAc-), Lewis Y (Fucα1-2Galß1-4(Fucα1-3)GlcNAc-) or lactosamine (Galß1-4GlcNAc) motifs. A comparative analysis of the glycan array data obtained for the recombinant full length ArathEULS3 protein (containing an additional *N*-terminal domain next to the EUL domain) as well as for the EUL domain revealed no major differences, confirming that the *N*-terminal domain of the chimeric protein does not interfere with glycan binding (Figure 3) [18].

Glycan array analysis of OrysaEULS2 protein from *Oryza sativa* confirmed the preferential binding of this EUL domain with mannosylated structures, as inferred from the agglutination inhibition assays. In contrast to ArathEULS3 and EEA, OrysaEULS2 shows a stronger interaction with high mannose *N*-glycans compared to the galactosylated structures (Figure 3). Indeed, the glycan array data revealed only very weak interaction of OrysaEULS2 with lactosamine structures (Figure 3) [28]. Since no binding of the lectin was observed to the high mannose structures lacking the chitobiose core, interaction of OrysaEULS2 with the glycan most probably requires the chitobiose core. 

The glycan array results for the EUL domain of PhypaEULS3 demonstrated that similar to ArathEULS3, PhypaEULS3 preferably binds to galactosylated carbohydrate structures. PhypaEULS3 strongly interacts with both *N*- and *O*-glycans containing lactosamine motifs (type I LacNAc and type II LacNAc), but it also recognizes glycans containing Lewis A, Lewis X and Lewis Y structures. The domain has only weak affinity for the blood group B related structures such as the glycans carrying the B-antigen, A-antigen and H-antigen structures (Figure 3). 

The results of the glycan array data also show that in contrast to EEA the binding of ArathEULS3, OrysaEULS2 and PhypaEULS3 to the array was much weaker (as reflected by the lower relative fluorescence unit values). Although this result is in agreement with the lower agglutination activity of these recombinant proteins, it cannot be excluded that the recombinant proteins expressed in the fungal host *Pichia pastoris* show a lower reactivity compared to the native lectins.

## 4. Three-dimensional Conformation of EUL Domains

**Figure 4:**
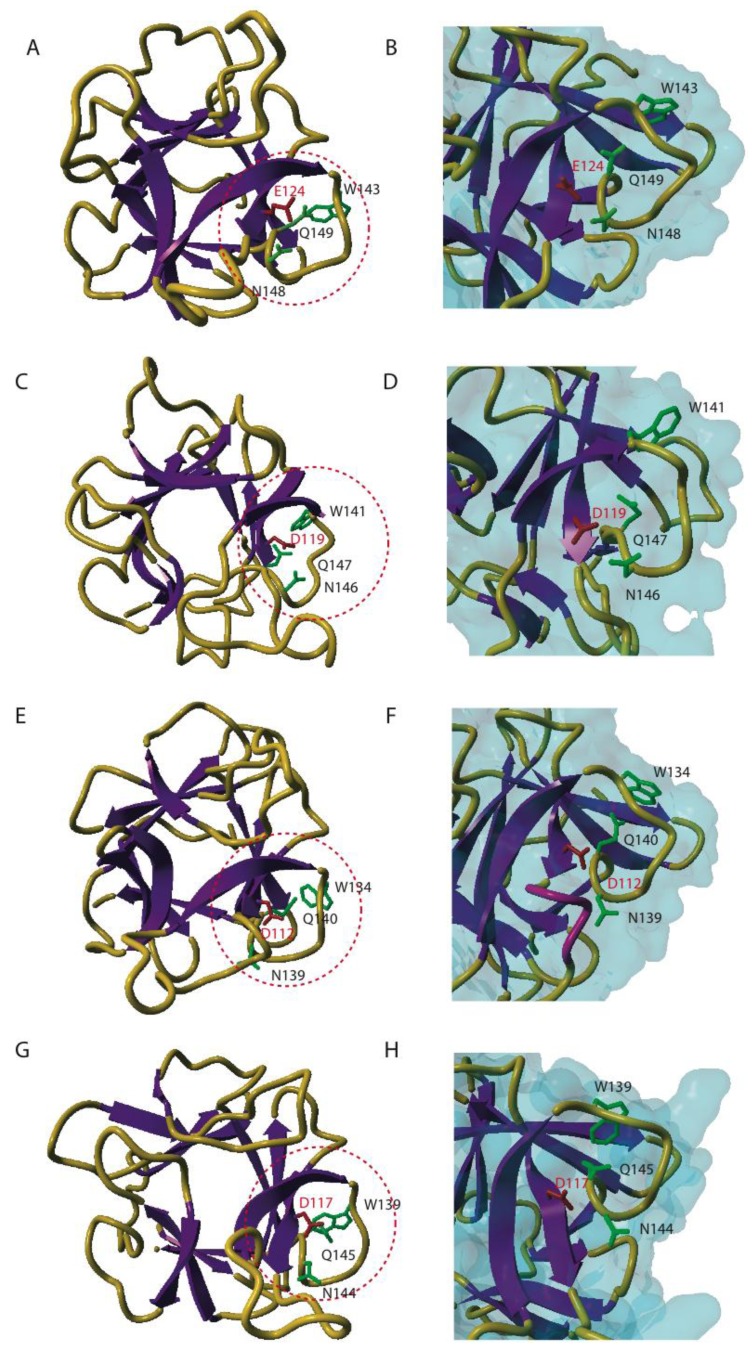
Three-dimensional models for different EUL domains. Ribbon diagrams of EEA (A) ArathEULS3 (C), OrysaEULS2 (E) and PhypaEULS3 (G) are shown. The strands of ß-sheet and the loops and coil regions are colored purple and orange yellow, respectively. The amino acid residues predicted to form the carbohydrate-binding site in a loop located at the *C*-terminal end of the polypeptide chain (red circle) are represented in green (W, N and Q residues) and red (E and D residues) sticks and labeled (according to the sequence). Panels B, D, F and H show an enlarged view of the carbohydrate-binding sites on the surface of EEA (B), ArathEULS3 (D), OrysaEULS2 (F) and PhypaEULS3 (H). The four amino acid residues forming the carbohydrate-binding sites are represented in sticks and labeled. The loop masking Asn139 residue in OrysaEULS2 is colored pink.

In an attempt to unravel which amino acids are required for the carbohydrate-binding activity of the EUL domain, three-dimensional models were made for the EUL domains in EEA, ArathEULS3, OrysaEULS2 and PhypaEULS3. Molecular modeling of the EUL domains was performed with the YASARA Structure program [34] and took advantage of the acceptable percentages of identity and homology between the different EUL sequences and the X-ray three-dimensional structures for the *C*-terminal domain of the HA33/A protein from *Clostridium botulinum* [35], the holotoxin from *Bacillus sphaericus* (RCSB PDB code 2VSE) [36], the *Sclerotinia sclerotiorum* agglutinin (RCSB PDB code 2X2S) [37] as well as the X-ray coordinates of a designed 3-fold symmetric protein (RCSB PDB code 3PG0) [38]. The use of these X-ray structures as a template allowed building accurate three-dimensional models for the different EUL domains (Figure 4). A comparison of the three-dimensional models built for EEA (Figure 4A) and the EUL domains of ArathEULS3 (Figure 4C), OrysaEULS2 (Figure 4E) and PhypaEULS3 (Figure 4G) revealed that they are all highly similar and represent a ß-trefoil fold consisting of three bundles of ß-sheet organized around a pseudo three-fold symmetry axis, connected by hairpin shaped loops.

The EUL sequences encoding EEA and ArathEULS3 both contain 1 cysteine (Cys) residue (Cys16 in EEA and Cys57 in ArathEULS3, Figure 2). Since Cys16 in EEA is exposed in a loop at the *N*-terminal end of the polypeptide chain it could create a disulphide bridge with the corresponding Cys residue of another ß-trefoil domain, resulting in the dimeric structure of EEA [14]. This dimer formation was observed for EEA after analysis of the purified protein by SDS-PAGE suggesting that this disulphide bridge formation occurs *in vitro*. In view of the biosynthesis of the lectin in the cytoplasmic compartment the bridging of cysteine residues is unlikely to occur *in vivo* since the required enzyme resides in the endoplasmic reticulum. The EUL sequences for OrysaEULS2 and PhypaEULS3 contain three and four Cys residues, respectively (Cys3, Cys100 and Cys135 in OrysaEULS2, and Cys7, Cys77, Cys104 and Cys105 in PhypaEULS3). Judging from the three-dimensional models, these Cys residues are too far from each other to create intra-chain disulphide bonds. However, these Cys residues are also sufficiently exposed to participate in inter-chain disulphide bonds with another lectin-like monomer to create homodimers. 

By comparison with the well identified carbohydrate-binding sites from other ß-trefoil sugar recognition domains of bacterial lectins of the ricin B family and, especially the HA33/A protein from *C. botulinum* (PDB code 1YBI) [35] a putative carbohydrate-binding site consisting of four well conserved residues was predicted at the *C*-terminal end of the four studied EUL proteins (Glu124, Trp143, Asn148, Gln149 for EEA; Asp119, Trp141, Asn146, Gln147 for the EUL domain in ArathEULS3; Asp112, Trp134, Asn139, Gln140 for the EUL domain in OrysaEULS2; Asp117, Trp139, Asn144, Gln145 for the EUL domain in PhypaEULS3) (Figure 2; Figure 4B, D, F, H). All residues forming the putative carbohydrate-binding site of the EUL domain are extremely conserved (Figure 2) and putative carbohydrate-binding sites appear as a charged groove as shown from the mapping of the electrostatic potentials on the molecular surface of the EUL domains [28]. An aromatic residue located in the vicinity of the putative carbohydrate-binding site e.g. Tyr147 in EEA and Trp132, Trp139 and Phe137 in the EUL domains of ArathEULS3, OrysaEULS2 and PhypaEULS3, respectively, probably participates in stacking interactions that are known to reinforce the binding of a simple sugar to the carbohydrate-binding site in plant lectins [39]. For OrysaEULS2 the position of the carbohydrate-binding site was validated by mutational analysis. Therefore different recombinant OrysaEULS2 proteins were synthesized in which one, two or three amino acids in the putative carbohydrate-binding site were mutated. All these mutant forms of OrysaEULS2 showed a strongly reduced binding to the glycan array compared to the original protein. Since mutation of Trp134 into Leu134 resulted in an almost complete loss of the carbohydrate-binding activity of OrysaEULS2, it was concluded that this amino acid plays an important role in the configuration of a functional carbohydrate-binding site [28].

## 5. Discussion

Evidence is accumulating that shows that the EUL domain represents a conserved structural unit of a family of carbohydrate-binding proteins that is widespread among plants. In this review the biological activity of EUL proteins from three plant species, in particular a monocot (*Oryza sativa*), a dicot (*Arabidopsis thaliana*) and a lower plant (*Physcomitrella patens*), were compared with EEA, the prototype of the EUL family.

### 5.1. Promiscuity of the EUL Binding Site

Analyses of the protein sequences of different members of this family showed that the EUL domain is strongly conserved in different plant species, whereas the *N*-terminal domains of the EUL proteins showed very little sequence homology. Despite the strong conservation of the EUL sequence, glycan array analyses showed that the specificity of the EUL domain ranges from high mannose *N*-glycans to blood-group B related structures and galactosylated epitopes [14,18,28]. Since all EUL proteins under study interact with glycans, it can be concluded that the EUL domain is a functional carbohydrate-binding domain. Nevertheless, the analyses of the specificity show that the carbohydrate-binding sites have evolved into recognition of different glycan structures. Clearly, gene divergence within the EUL family leads to changes in carbohydrate-binding specificity. This promiscuity of the sugar-binding site is not unique to EUL proteins. In other lectin families it was also shown that gene divergence results in deviations of carbohydrate-binding specificity. For instance, within the legume lectin family, which consists of proteins with a high similarity of amino acid sequences and tertiary structures, it was shown that different mono and oligosaccharides can be selected by a conserved scaffold. It appears that a basic set of essential and conserved residues is surrounded by a limited number of variable amino acid residues that direct the specificity of the lectin [40,41]. Similarly, glycan microarray analyses of the *Galanthus nivalis* agglutinin (GNA) and a homologous protein from maize which shares 64% sequence similarity and has conserved residues in the carbohydrate-binding sites, showed important differences in their specificity. While GNA binds strongly to high mannose *N*-glycans the GNA homologue from maize has a high affinity for more complex glycans [42]. Recently, plasticity in the carbohydrate-binding site was also reported for lectins comprising a Nictaba domain. Nictaba, a jasmonate inducible lectin in tobacco leaves interacts preferentially with GlcNAc oligomers and high mannose and complex *N*-glycans [12]. Although there is a high sequence similarity between Nictaba and the *C*-terminal Nictaba domain of the so-called F-box Nictaba proteins, glycan array analysis clearly demonstrated that the *Arabidopsis* F-box protein exhibits a substantially different carbohydrate-binding specificity [43]. *N*- and *O*-glycans containing LacNAc structures as well as Lewis A, Lewis X, Lewis Y and blood type B motifs were recognized by the Nictaba domain of the *Arabidopsis* F-box protein. All these data show that similar carbohydrate-binding motifs can accommodate unrelated oligosaccharides.

Molecular modeling of the EUL domains of EEA, ArathEULS3, OrysaEULS2 and PhypaEULS3 demonstrated that they all exhibit a similar three-dimensional structure which is a ß-trefoil fold consisting of three bundles of ß-sheet. Although the EUL proteins show a very similar overall fold, there are clear differences in the overall structure and the charge distribution on the protein surface. It was shown before that small changes in the amino acids building the carbohydrate-binding site or surrounding the site can lead to changes in their specificity [39]. For instance, the positioning of Asn139 in the EUL domain of OrysaEULS2 can influence the carbohydrate-binding properties of this protein. This amino acid, important for the sugar-binding properties in OrysaEULS2 is apparently masked by an extended loop that protrudes in the vicinity of the carbohydrate-binding site and thus this residue should no longer be available for a hydrogen bond interaction with the sugar [28]. This can also be an explanation for the reduced binding of OrysaEULS2 with the glycans on the array compared to the binding of EEA. 

Similarly, a single variable loop determines the exact shape of the promiscuous monosaccharide binding site of the legume lectins and is responsible for discrimination between galactose, mannose or glucose. Lectin II from *Ulex europaeus* (UEA-II), a legume lectin, has a binding site which is capable of binding both *N*-acetylglucosamine and galactose. It was shown that hydrophobic interactions are important in the protein–carbohydrate complexes [40].

### 5.2. Physiological Relevance of Carbohydrate-Binding Activity of EUL Lectins

At present, one can only speculate about the physiological role of proteins containing an EUL domain. Taking into consideration that all proteins with an EUL domain are synthesized in the cytoplasm and that the EUL domain apparently possesses lectin activity, it seems reasonable to expect that the biological activity of these proteins relies on their binding to cytoplasmic/nuclear receptors. In principle, both free *N*-glycans (resulting from de novo synthesis or *N*-glycoconjugate degradation [44,45]) as well as glycans attached to glycoproteins are potential targets for these lectins. The question arises whether suitable glycosylated receptors can be found in the nucleocytoplasmic compartment. Unfortunately, there is limited conclusive evidence at the moment for the occurrence of glycosylated proteins in the nucleus and the cytoplasm. In principle, cytoplasmic lectins could also bind to free metabolic glycans, such as cytosolic heteroglycans resulting from the degradation of leaf starch [46,47], but until now this interaction has never been studied.

It was shown that EEA and OrysaEULS2 have affinity for high mannose type *N*-glycans. As a consequence high mannose containing free *N*-glycans as well as glycosylated proteins are putative targets for these lectins. Free *N*-glycans are reported to occur ubiquitously at micromolar levels in various cells or tissues like in elongating hypocotyls of seedlings, developing seeds or maturated fruits, indicating that these free *N*-glycans play an important role during cell differentiation, plant growth or fruit ripening [44,45,48,49,50,51]. Two types of free *N*-glycans, a high mannose type and a complex type, were found. A quantitative analysis of high mannose type free *N*-glycans using cotyledons of pumpkin seedlings as model cells showed that the high mannose free *N*-glycans accumulate in the cytosol [52]. Although it is not yet clear whether free *N*-glycans act as signaling molecules, it appears likely that the *N*-glycosylation mechanisms working in plants are associated with plant development, plant growth or fruit ripening. 

Next to high mannose *N*-glycans, galactosylated structures were recognized by several EUL proteins. EEA strongly binds to blood group B related structures. Glycan array analysis revealed that the EUL protein from the lower plant *Physcomitrella patens* has a high affinity for glycans containing lactosamine structures. These LacNAc structures were also recognized by the EUL from the dicot *Arabidopsis thaliana* and the monocot *Oryza sativa*. PhypaEULS3 binds also Lewis A structures while ArathEULS2 recognizes Lewis X and Lewis Y structures. 

EULs are not the only lectins that recognize galactosylated structures. The family of human galectins for instance also has specificity towards the disaccharide LacNAc, poly-LacNAc and internal LacNAc present in poly-LacNAc [53]. Moreover, similar to the EUL proteins several members of the galectin family are located in the nucleus and the cytoplasm of the cell [54]. Also the nucleocytoplasmic plant lectin F-box-Nictaba recognizes LacNAc motifs [42].

Although Lewis X and Lewis Y structures can be found in higher animals they have not yet been identified in plants. Until now only Lewis A motifs have been reported at the non-reducing end of biantennary complex type plant *N*-glycans [55,56,57]. These Lewis A epitopes were also detected in *N*-glycans of a glycoallergen from mountain cedar pollen [58,59]. Structural analysis of the *N*-glycans linked to glycoproteins in rice cells revealed that most of them had the plant complex type structure including a Lewis A epitope-harboring type whereas the high mannose type structures were low abundant [60]. It was shown more than two decades ago that free *N*-glycans could also be secreted [61]. In 2010, Maeda *et al*. [62], analyzed the free *N*-glycans in the intracellular and the extracellular spaces of a rice cell culture system. It was observed that the sugar chain profile differed between the soluble fraction and the cell culture medium of rice-cultured cells. The intracellular fraction contained mainly high mannose type *N*-glycans with one GlcNAc residue while in the culture medium complex free *N*-glycans containing the Lewis A epitope and high mannose type free *N*-glycans with the *N*-diacetylchitobiosyl unit were found [61]. Secretion of glycoproteins containing Lewis A motifs could suggest a putative role in stress signaling or cell-cell communication as suggested before [63].

Next to the carbohydrate-binding properties of the EUL proteins, their presence in low concentrations in the plant cell under specific stress conditions also urges for a specific role of these lectins within the plant cell. However, in contrast to ArathEULS3, OrysaEULS2 and PhypaEULS3 EEA is an abundant protein in the arillus where it accounts for up to 80% of the total protein content. Besides in the arilli, the lectin is also found in leaves and bark tissue, though at much lower concentrations [15]. No lectin has been detected in the seeds. Taking into account the preferential accumulation of the lectin at an extremely high level in the specialized arillus tissue, EEA can hardly play an essential role in the plant itself. On the analogy of other plant lectins that accumulate at high concentrations, a role as defence and/or storage-related protein seems more likely [4,10]. However, since the arillus tissue cannot be considered a storage tissue but rapidly decays as soon as the seeds are shed into the litter under the trees, it is evident that EEA does not act as a storage protein. Therefore the only plausible explanation is that the massive accumulation of EEA protects the developing arillus against potential phytophagous invertebrates and/or herbivorous animals. In this respect, EEA definitely differs from the EUL proteins found in rice, which are expressed at much lower levels and only in response to a specific stress condition [19,64]. Most probably the highly expressed EEA represents a specialized form that diverged from the main evolutionary line by an evolutionary event whereby a gene encoding a stress-related lectin was duplicated and placed under the control of a novel promoter. The narrow taxonomic distribution of highly abundant EEA-like lectins supports the occurrence of such an evolutionary event [9]. It has been demonstrated before that e.g. in the family of jacalin-related lectins, the lectin from *Calystegia sepium* rhizomes is classified as a mannose-binding jacalin-related lectin [65]. In contrast to most mannose-binding lectins related to jacalin, Calsepa is a very abundant protein, but similar to other mannose-binding jacalin-related lectins it is also located in the cytoplasm. Its high concentration and exclusive accumulation in a vegetative storage tissue indicate that the *Calystegia sepium* lectin is just a cytoplasmic storage and/or defence related protein [66].

## 6. Conclusions

This evidence shows that the EUL domain can be considered a universal lectin domain within the plant kingdom. However, it is clear that the specificity of the binding site has evolved during evolution. It can be concluded that it is difficult to make a prediction on the specificity of the EUL domain based on the sequence and on the conservation of the amino acids constituting the sugar-binding site. Surely, a detailed analysis of the carbohydrate-binding specificity of more members of the EUL family will provide more insight as to their specificity. Since these carbohydrate-binding properties most likely are a determining factor for their physiological role in the plant cell, this knowledge will also help to unravel the biological significance of these proteins. Future work will focus on the identification of the interacting partners of the EUL lectins with the ultimate goal of unraveling their physiological importance for plant growth and development.
